# Deep brain stimulation of the anterior nuclei of the thalamus relieves basal ganglia dysfunction in monkeys with temporal lobe epilepsy

**DOI:** 10.1111/cns.13462

**Published:** 2020-10-21

**Authors:** Tingting Du, Yingchuan Chen, Lin Shi, Defeng Liu, Yuye Liu, Tianshuo Yuan, Xin Zhang, Guanyu Zhu, Jianguo Zhang

**Affiliations:** ^1^ Department of Functional Neurosurgery Beijing Neurosurgical Institute Capital Medical University Beijing China; ^2^ Department of Neurosurgery Beijing Tiantan Hospital Capital Medical University Beijing China; ^3^ Beijing Key Laboratory of Neurostimulation Beijing China

**Keywords:** anterior nuclei of the thalamus, basal ganglia, deep brain stimulation, epilepsy, neuroprotection

## Abstract

**Aims:**

Deep brain stimulation of the anterior nuclei of the thalamus (ANT‐DBS) is effective in temporal lobe epilepsy (TLE). Previous studies have shown that the basal ganglia are involved in seizure propagation in TLE, but the effects of ANT‐DBS on the basal ganglia have not been clarified.

**Methods:**

ANT‐DBS was applied to monkeys with kainic acid–induced TLE using a robot‐assisted system. Behavior was monitored continuously. Immunofluorescence analysis and Western blotting were used to estimate protein expression levels in the basal ganglia and the effects of ANT stimulation.

**Results:**

The seizure frequency decreased after ANT‐DBS. D1 and D2 receptor levels in the putamen and caudate were significantly higher in the ANT‐DBS group than in the epilepsy (EP) model. Neuronal loss and apoptosis were less severe in the ANT‐DBS group. Glutamate receptor 1 (GluR1) in the nucleus accumbens (NAc) shell and globus pallidus internus (GPi) increased in the EP group but decreased after ANT‐DBS. γ‐Aminobutyric acid receptor A (GABA_A_‐R) decreased and glutamate decarboxylase 67 (GAD67) increased in the GPi of the EP group, whereas the reverse tendencies were observed after ANT‐DBS.

**Conclusion:**

ANT‐DBS exerts neuroprotective effects on the caudate and putamen, enhances D1 and D2 receptor expression, and downregulates GPi overactivation, which enhanced the antiepileptic function of the basal ganglia.

## Graphical abstract

ANT‐DBS exerts neuroprotective effects on the caudate and putamen, enhances D1 and D2 receptor expression, and downregulates GPi overactivation, which enhanced the antiepileptic effect of the basal ganglia.

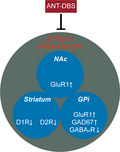

## 1 INTRODUCTION

Epilepsy is a chronic and disabling disease that afflicts 4‐10 individuals per 1000.^1^ One‐third of patients do not achieve seizure control with antiepileptic drugs, particularly those with mesial temporal lobe epilepsy (TLE).^2^ Deep brain stimulation of the anterior nuclei of the thalamus (ANT‐DBS) is now one of the most frequently used neuromodulation therapies for patients with drug‐resistant TLE. The efficacy of ANT‐DBS has been confirmed by a randomized, multicenter, double‐blind SANTE trial, and the US Food and Drug Administration approved this treatment for epilepsy in 2018.^3^, ^4^

Hippocampal neuron loss and limbic circuit reformation are the most important pathogenic features of TLE.^3^ The basal ganglia system interacts with the limbic system and could be affected in TLE. One study identified atrophy of the basal ganglia in patients with TLE, but not in patients with extratemporal foci.^5^ Another study demonstrated reduced D2 receptors in the striatal and extrastriatal regions of TLE patients with positron emission tomography (PET).^6^ Gale *et al* proposed that the substantia nigra pars reticulata (SNr) of rodents influences the widespread cerebral mechanisms involved in the synchronization and excitability that occur in epilepsy.^7^ Previous studies have also found that inhibition of the SNr inhibits epileptogenesis.^8^, ^9^

The studies cited above suggest that the basal ganglia are involved in TLE, but the mechanism is still unclear. The limbic and basal ganglia systems have mainly been investigated in human and nonhuman primate (NHP) studies, but the role of the basal ganglia circuit in TLE has been investigated less extensively.^10^ Importantly, only in primates can the pallidus be divided into the external globus pallidus (GPe) and the internal globus pallidus (GPi). The complex circuitry of the basal ganglia and the cortex is also unique to primates.^11^ Therefore, use of NHPs is important when assessing basal ganglia circuit changes in TLE and after ANT‐DBS.

We used a robot‐assisted ANT‐DBS implantation platform and well‐characterized kainic acid–induced model of epilepsy to investigate basal ganglia changes in NHP‐TLE after ANT‐DBS. The main basal ganglia circuit structure, which includes the nucleus accumbens (NAc), dorsal striatum (caudate and putamen), and GPi, was investigated in this study.

## 2 MATERIALS AND METHODS

### 2.1 Animals and ethics

Twenty‐four male rhesus macaques (provided by the Animal Center of Military Medical Sciences, Beijing, China; age, 7.3 ± 1.2 years; weight, 8.3 ± 1.4 kg) were randomly divided into a control group (n = 6), epilepsy (EP) group (n = 6), EP‐sham‐DBS group (n = 6), and EP‐DBS group (n = 6). All the animals were raised in an environmentally controlled room (23‐25°C, 12‐h light/12‐h dark cycle, lights on at 07:00) with free access to food and water. The experiments were performed in accordance with the Guidelines for the Use and Care of Experimental Animals. This study was approved by the Ethics Committee of Beijing Neurosurgical Institute, Beijing, China (no. 201 703 002). All efforts were made to minimize animal suffering.

### 2.2 Establishment of the epileptic model and behavior monitoring

The epileptic model was established according to our previous study.^12^ Briefly, all the monkeys were subjected to general anesthesia with an intramuscular injection of Zoletil (5 mg/kg; Virbac, Alpes‐Maritimes, France) and Dexdomitor (20 μg/kg; Zoetis, Parsippany, NJ, USA), before a magnetic resonance imaging (MRI) scan with a 3‐Tesla MRI scanner (Sigma; GE Healthcare, Waukesha, WI, USA). Kainic acid or normal saline was injected into the monkeys on the workstation of a neurosurgical robot system (RM‐100; Beijing Baihui Weikang Technology Co., Ltd, Beijing, China), according to the surgical plan based on preoperative MRI. In the EP, EP‐sham‐DBS, and EP‐DBS groups, kainic acid (1 μg/μL; 1.5 μg/kg/target) was injected into the left hippocampus and amygdala according to the surgical plan, and normal saline (1.5 μL/kg/target) was injected into the same points in the control group animals. The vital signs of the animals were monitored throughout surgery.

Stereoelectroencephalography (SEEG) electrodes (SDE‐08; Beijing HKHS Healthcare Co. Ltd., Beijing, China) were implanted into the left hippocampus at 24 h and 1 month after injection to detect epileptic discharge. Monitoring was sustained for 2 h under anesthesia, as described in our previous study.^12^ Status epilepticus was defined as a single epileptic seizure lasting > 5 min or two or more seizures within a 5‐min period without the monkey returning to normal between them. The behavior of all the animals was monitored throughout the experiment, and only the seizures that occurred between 4 weeks after injection and the end of the experiment were used for comparison.^13^

### 2.3 ANT‐DBS implantation and stimulation

Three weeks after the kainic acid injection, the monkeys in the EP‐sham‐DBS and EP‐DBS groups were implanted with ANT‐DBS electrodes. The electrodes (L301; Beijing PINS Medical Co. Ltd, Beijing, China; diameter: 1.27 mm; contact length: 1.5 mm; contact space: 0.5 mm; contact number: 4) were designed to target the left ANT based on the individuals’ MRI scans and an atlas of the monkey brain.^14^ An extension was tunneled subcutaneously through the neck to the abdomen, where an implantable pulse generator (IPG, G102, Beijing PINS Medical Co. Ltd) was located. Postoperative computed tomography (CT) was performed to detect any complications and to confirm accurate placement of the lead. One week after lead implantation, the monkeys in the EP‐DBS group were stimulated for 8 weeks (stimulation parameters: 1.5 V, 90 μs, and 150 Hz), with sites of stimulation contact selected based on fused postoperative images, whereas no electric stimulation was delivered to animals in the EP‐sham‐DBS group.

The lead position errors were measured by fusing the images from preoperative MRI and postoperative CT and comparing them with the surgical plan. These data have been reported in our previous study and indicated accurate lead positions.^15^

### 2.4 Tissue processing

**FIGURE 1:**
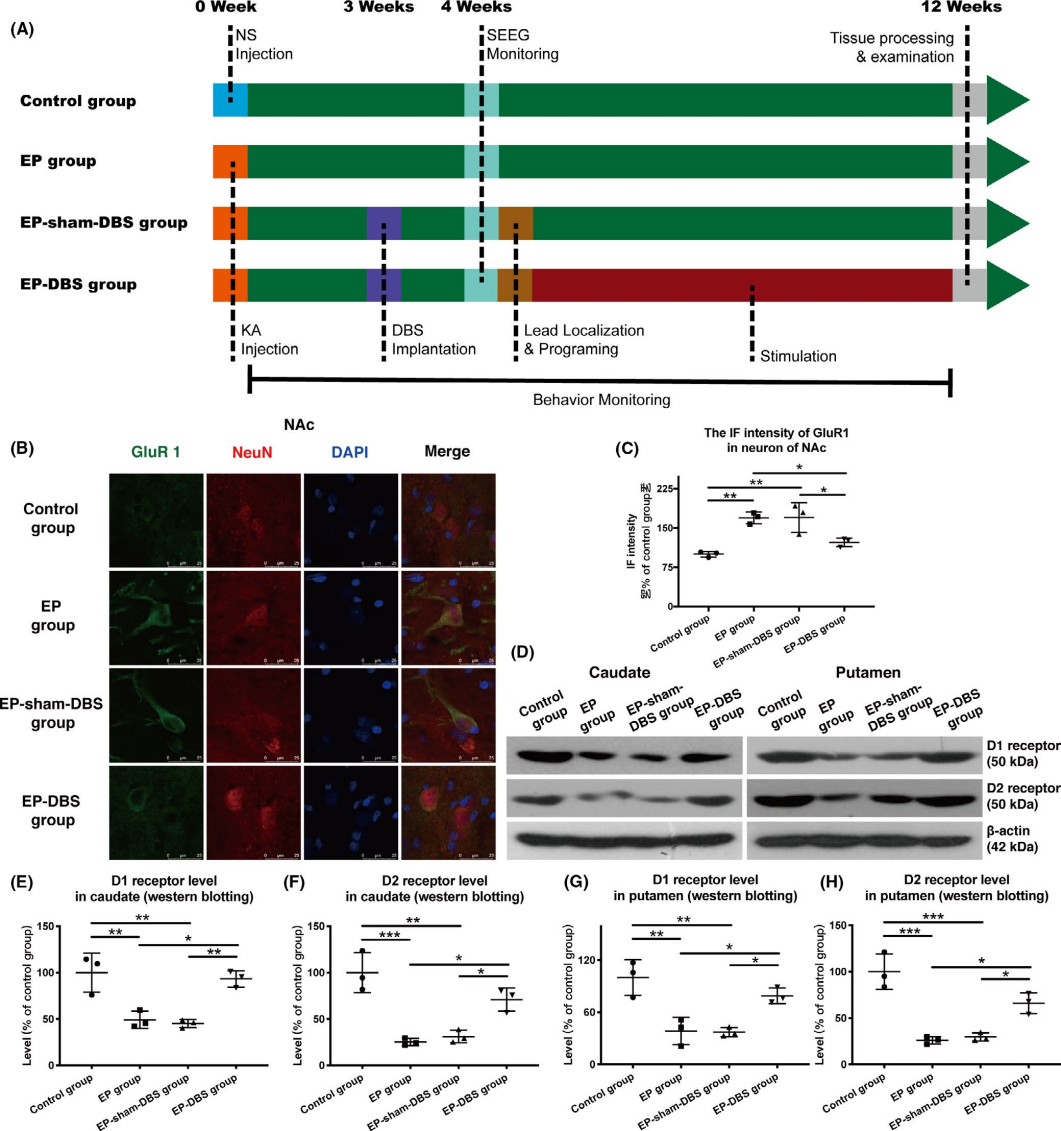
A, Roadmap of the research. Twenty‐four monkeys were randomly assigned to the control, EP, EP‐sham‐DBS, and EP‐DBS groups. Different manipulations were applied to the four groups, but only the monkeys in the EP‐DBS group received ANT stimulation for 2 months. Different colors indicate different time points (blue: NS injection; orange: KA injection; purple: DBS implantation; cyan, SEEG monitoring; brown: lead localization and programming; red: stimulation). B, Immunofluorescent staining of GluR1 and NeuN in NAc shell. C, High immunofluorescence intensity of GluR1 was observed in the NAc shell of animals in the EP and EP‐sham‐DBS groups. However, ANT‐DBS normalized the expression of GluR1. D, Analysis of D1 and D2 receptors in caudate and putamen with Western blotting. E–H, In the EP monkey model, D1 and D2 receptor levels declined in both the caudate and putamen. Expression of D1 and D2 receptors in the caudate and putamen was markedly higher in the EP‐DBS group than in the EP or EP‐sham‐DBS group. *P < .05; **P < .01; ***P < .001. DBS, deep brain stimulation; ANT, anterior nuclei of the thalamus; EP, epilepsy; GluR1, glutamate receptor 1; NAc, nucleus accumbens [Colour figure can be viewed at wileyonlinelibrary.com]

After stimulation for 8 weeks or not, all the monkeys were deeply anesthetized and sacrificed. Three animals in each group were randomly selected for fresh tissue processing (subgroup A), and the left caudate, putamen, and GPi were removed and stored at −80°C. The remaining three monkeys in each group (subgroup B) were perfused with normal saline and then with 4% paraformaldehyde in 0.1 mol/L phosphate‐buffered saline (PBS) before the brain was removed (Figure 1A).

### 2.5 Western blotting analysis

The brain tissues were washed with PBS and lysed in radioimmunoprecipitation assay buffer (50 mM Tris‐HCl [pH 7.4], 150 mM sodium chloride, 1% Nonidet P‐40, 0.1% sodium dodecyl sulfate [SDS]), containing phosphate and protease inhibitor cocktails. The homogenates were centrifuged at 12,000 × g for 20 min. Protein concentrations in the supernatants were determined with a bicinchoninic acid protein assay kit (Pierce, Rockford, IL, USA). Total protein (60 μg) was resolved with SDS‐polyacrylamide gel electrophoresis on a 12% polyacrylamide gel and transferred to polyvinylidene difluoride membrane (Millipore, Billerica, MA, USA). After the membranes were blocked with 10% milk for 1 h, they were incubated with the following primary antibodies: rabbit anti‐β‐actin (1:5000; Sigma‐Aldrich, A5060), mouse anti‐NeuN (1:500; Millipore, MAB377), rabbit anti‐glutamate receptor 1 (GluR1; 1:500; abcam, ab31232), rat anti‐dopamine D1 receptor (1:500; Sigma‐Aldrich, D2944), rabbit anti‐dopamine D2 receptor (1:500; Millipore, AB5084P), mouse anti‐glutamate decarboxylase 67 (GAD67; 1:1000; Invitrogen, MA5‐24909), rabbit anti‐cleaved‐caspase 3 (1:1000; Cell Signaling Technology, 9661), and rabbit anti‐GABA_A_‐R alpha 1 (1:500; abcam, ab33299). The membranes were incubated with a secondary antibody, and the protein bands were visualized with enhanced chemiluminescence and quantified using ImageJ software. β‐Actin was used as the loading control. Uncropped images of the Western blots are shown in Figure S1.

### 2.6 Immunofluorescent (IF) staining

IF was performed as described in a previous study.^16^ Briefly, brain tissue sections were rinsed in PBS and permeabilized with 0.3% Triton X‐100 in PBS for 30 min at room temperature. After the sections were blocked with 10% normal goat serum for 1 h, they were incubated overnight at 4°C with mouse anti‐NeuN (1:100; Millipore, MAB377), rabbit anti‐NeuN (1:500; abcam, ab128886), rabbit anti‐GluR1 (1:1000; abcam, ab31232), rat anti‐dopamine D1 receptor (1:100; Sigma‐Aldrich, D2944), rabbit anti‐dopamine D2 receptor (1:100; Millipore, AB5084P), mouse anti‐GAD67 (1:50; Invitrogen, MA5‐24909), or rabbit anti‐GABA_A_‐R alpha 1 (1:50; abcam, ab33299) antibody, followed by an Alexa‐Fluor‐647‐, −594‐, or −488‐conjugated secondary antibody (1:500; Life Technologies) for 1 h at room temperature. Cell nuclei were visualized by counterstaining them with 4′,6‐diamidino‐2‐phenylindole (DAPI; Sigma‐Aldrich). The sections were observed via confocal microscopy (TCS SP8; Leica, Solms, Germany).

Five matched sections (equally spaced from the anterior to the posterior part of the nuclei) from each group were prepared for evaluation based on the atlas of the monkey brain ^14^ (GPi: 13‐19 mm [anterior from the atlas‐based basal plane], at an approximate interval of 1.5 mm; 18‐30 mm [anterior from the atlas‐based basal plane], at an approximate interval of 3 mm; 10‐22 mm [anterior from the atlas‐based basal plane], at an approximate interval of 3 mm), and six views of cells in each section were randomly selected for further measurement of the positive neuron number or IF intensity (according to the instructions in “The Open Lab Book” [https://theolb.readthedocs.io/en/latest/imaging/measuring‐cell‐fluorescence‐using‐imagej.html] using ImageJ software). The average value was taken as the value for each individual.

### 2.7 Statistical analysis

Data are expressed as the mean ± standard deviation (SD). The Shapiro‐Wilk test was used to evaluate the normality of the data. All *P*‐values for the Shapiro‐Wilk test were > 0.05. One‐way ANOVA followed by Bonferroni *post hoc* correction was used to analyze the statistical significance of differences among multiple groups. All statistical analyses were performed with SPSS 21.0 software (IBM, Chicago, IL, USA) and plotted using the GraphPad Prism version 6.00 software package (GraphPad Software). A *P*‐value of less than .05 was considered significant. *P*‐values for the different comparisons are summarized in Table S1.

## 3 RESULTS

### 3.1 Beneficial effect of ANT‐DBS in controlling seizure frequency

No seizures were observed in the control group, whereas status epilepticus and recurrent seizures were observed in all monkeys in the EP, EP‐sham‐DBS, and EP‐DBS groups. The total seizure number was significantly reduced by ANT‐DBS when compared to the EP group or EP‐sham‐DBS group, as shown in our previous study.^13^ Both partial and generalized seizures were recorded in these groups; detailed information can be found in our previous study.^13^ Abnormal discharge on SEEG was also observed in our previous study.^12^ We observed no severe side effects or abnormal responses among the groups.^13^

### 3.2 Expression of glutamate receptor on the NAc shell is reduced by ANT‐DBS

The IF intensity of GluR1 in the NAc shell was significantly higher in animals in the EP and EP‐sham‐DBS groups than in the control group. However, ANT‐DBS normalized the expression of GluR1 (control group 100.0 ± 5.2%, EP group 169.4 ± 11.2%, EP‐sham‐DBS group 170.0 ± 28.4%, EP‐DBS group 122.6 ± 8.1%, *F*_(3,8)_ = 14.32, *P* = .0014) (Figure 1B,C).

### 3.3 ANT‐DBS reduces dopamine receptor D1 and D2 loss in caudate and putamen

We used Western blotting to evaluate D1 and D2 receptor expression in the ipsilateral caudate and putamen. Relatively high and normal D1 and D2 receptor levels were observed in the control group. However, D1 receptor levels declined in EP and EP‐sham‐DBS groups. Markedly higher expression of D1 receptors was observed in the EP‐DBS group in comparison with the EP and EP‐sham‐DBS groups. A similar trend was observed for D2 receptor expression in the caudate and putamen (D1 caudate: control group 100.0 ± 20.9%, EP group 49.1 ± 9.3%, EP‐sham‐DBS group 45.2 ± 4.4%, EP‐DBS group 93.3 ± 8.9%, *F*_(3,8)_ = 15.88, *P* = .0010; D2 caudate: control group 100.0 ± 21.4%, EP group 25.3 ± 3.9%, EP‐sham‐DBS group 31.1 ± 6.9%, EP‐DBS group 70.9 ± 12.5%, *F*_(3,8)_ = 22.00, *P* = .0003); D1 putamen: control group 100.0 ± 20.7%, EP group 38.5 ± 15.7%, EP‐sham‐DBS group 37.2 ± 5.2%, EP‐DBS group 79.0 ± 8.9%, *F*_(3,8)_ = 14.80, *P* = .0013; D2 putamen: control group 100.0 ± 19.2%, EP group 26.1 ± 4.1%, EP‐sham‐DBS group 29.7 ± 4.4%, EP‐DBS group 66.1 ± 11.2%, *F*_(3,8)_ = 27.21, *P* = .0002) **(**Figure 1D–H).

**FIGURE 2:**
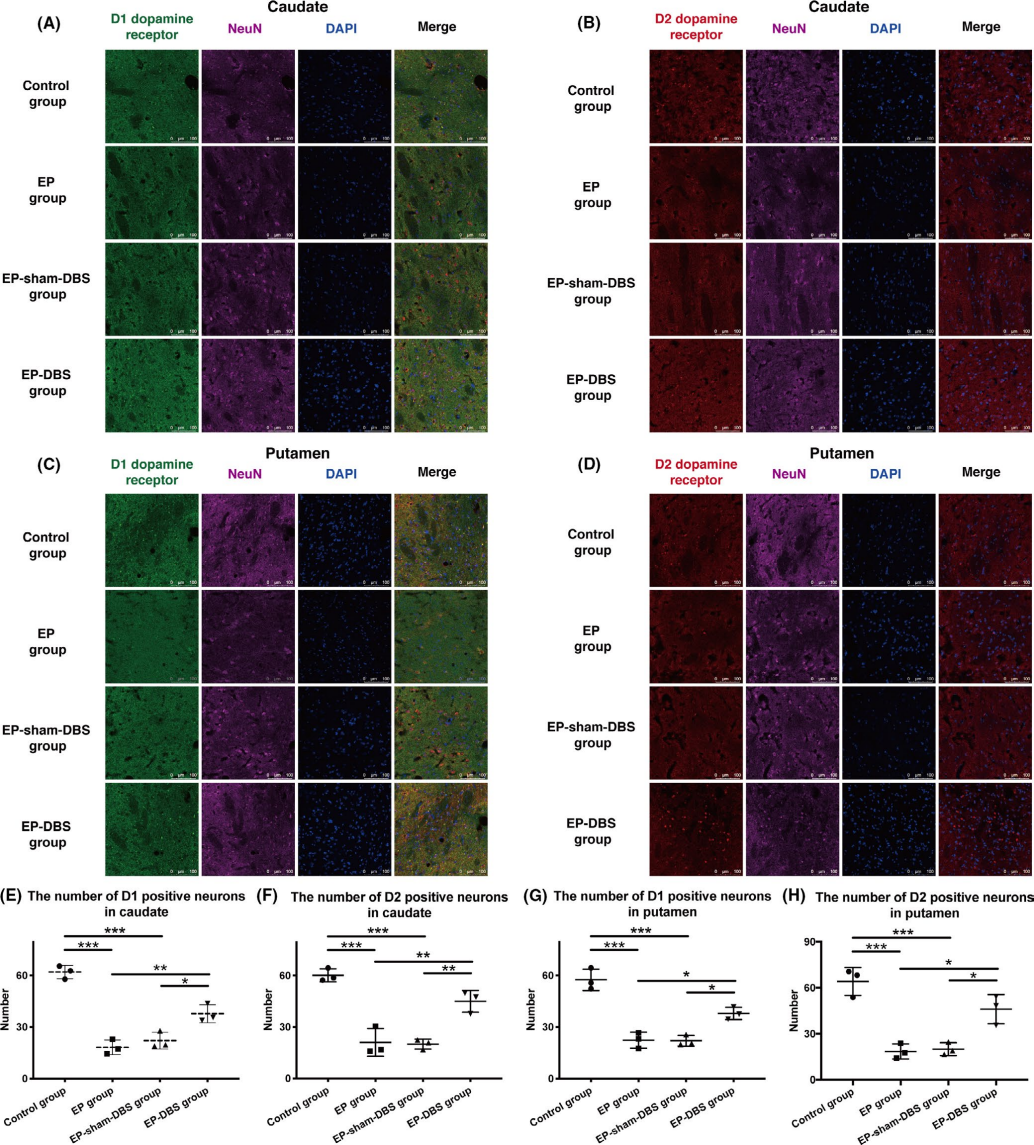
A and B, Immunofluorescent staining of D1 and D2 dopamine receptors and NeuN in the caudate. C and D, Immunofluorescent staining of D1 and D2 dopamine receptors and NeuN in the putamen. E–H, Numbers of D1 receptor‐ and D2 receptor‐positive neurons in the caudate and putamen were lower in the EP and EP‐sham‐DBS groups than in the normal monkeys, but these trends were reversed by ANT‐DBS. *P < .05; **P < .01; ***P < .001. DBS, deep brain stimulation; ANT, anterior nuclei of the thalamus; EP, epilepsy [Colour figure can be viewed at wileyonlinelibrary.com]

D1 and D2 receptors can be expressed in glia cells as well as in presynaptic neurons,^17^ so colocalization of D1 or D2 receptors and NeuN (a neuronal marker) was determined to specifically measure the number of D1‐ and D2‐positive neurons. Similar to the Western blotting results, the numbers of D1 receptor and D2 receptor‐positive neurons in the caudate and putamen declined sharply in the EP and EP‐sham‐DBS groups compared with the control group, whereas these trends were reversed by ANT‐DBS (D1 caudate: control group 62 ± 3.9, EP group 18.2 ± 4.2, EP‐sham‐DBS group 22.1 ± 5.0, EP‐DBS group 37.7 ± 5.2, *F*_(3,8)_ = 55.95, *P* < .0001; D2 caudate: control group 60.1 ± 3.8, EP group 21.0 ± 8.1, EP‐sham‐DBS group 20.0 ± 2.9, EP‐DBS group 45.0 ± 6.4, *F*_(3,8)_ = 35.17, *P* < .0001; D1 putamen: control group 57.5 ± 6.2, EP group 22.4 ± 4.7, EP‐sham‐DBS group 22.0 ± 3.1, EP‐DBS group 37.9 ± 3.6, *F*_(3,8)_ = 40.37, *P* < .0001; D2 putamen: control group 64.2 ± 9.1, EP group 18.5 ± 4.9, EP‐sham‐DBS group 20.0 ± 4.2, EP‐DBS group 46.1 ± 9.6, *F*_(3,8)_ = 26.85, *P* = .0002) (Figure 2A–H).

### 3.4 Neuron loss in the caudate and putamen is relieved by ANT‐DBS

**FIGURE 3:**
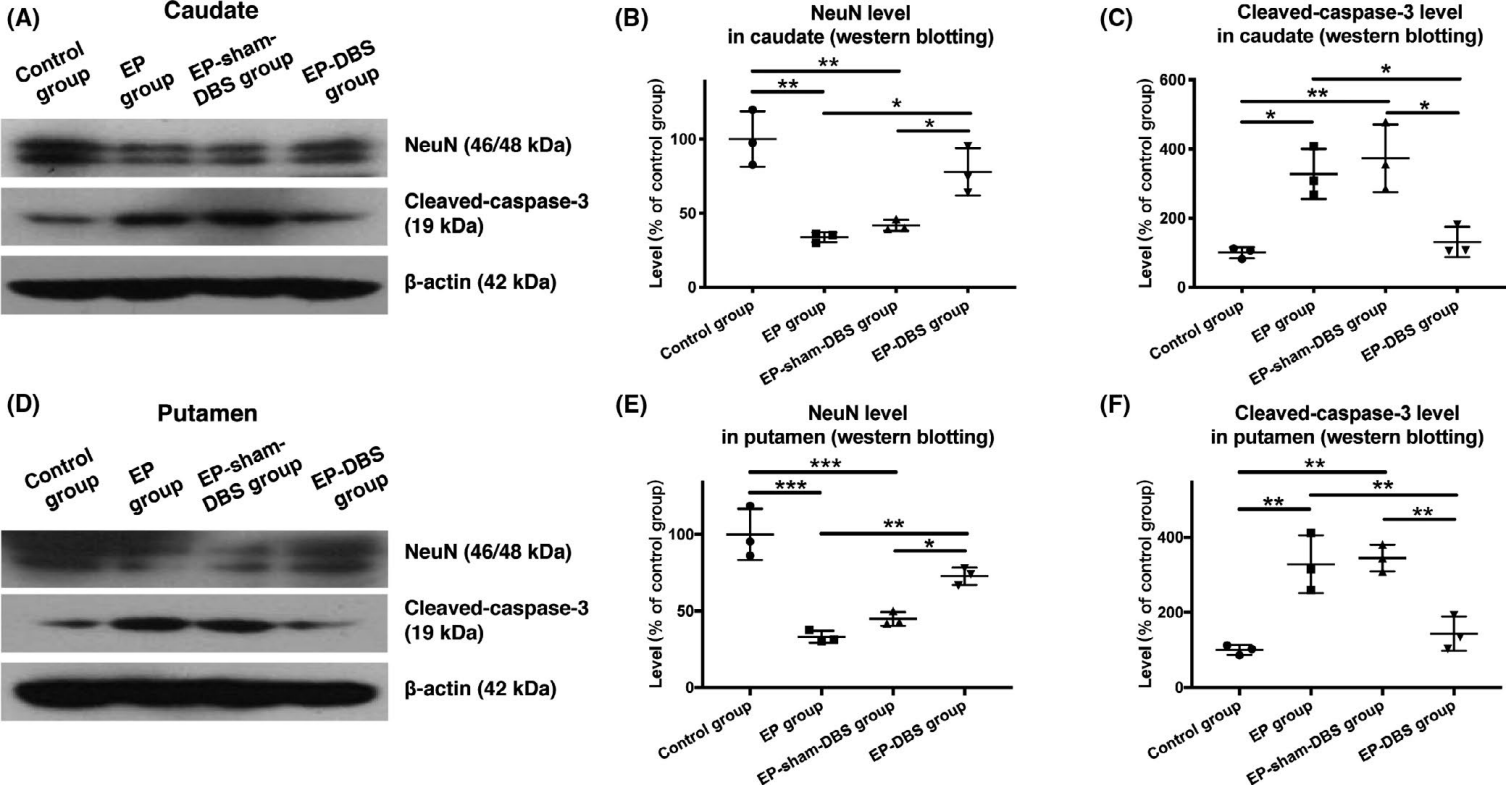
A and D, Analysis of NeuN and cleaved‐caspase 3 in the caudate and putamen via Western blotting. B and E, NeuN levels were significantly reduced in the caudate and putamen of the EP and EP‐sham‐DBS groups, but were higher in the caudate and putamen of the EP‐DBS group than in those of the EP and EP‐sham‐DBS groups. C and F, Levels of cleaved‐caspase 3 were higher in the caudate and putamen of EP and EP‐sham‐DBS groups than in those of the control group, but the expression of cleaved‐caspase 3 was lower in the caudate and putamen of the EP‐DBS group, compared with EP monkey model. *P < .05; **P < .01; ***P < .001. DBS, deep brain stimulation; EP, epilepsy

NeuN levels were significantly reduced in the caudate and putamen in the EP and EP‐sham‐DBS groups in comparison with the control group, but were higher in the caudate and putamen in the EP‐DBS group relative to those in the EP and EP‐sham‐DBS groups, indicating that more neurons survived after ANT‐DBS (caudate: control group 100.0 ± 18.7%, EP group 33.8 ± 3.3%, EP‐sham‐DBS group 41.8 ± 3.7%, EP‐DBS group 77.9 ± 16.0%, *F*_(3,8)_ = 18.32, *P* = .0006; putamen: control group 100.0 ± 16.7%, EP group 33.1 ± 3.9%, EP‐sham‐DBS group 44.8 ± 4.5%, EP‐DBS group 72.7 ± 5.7%, *F*_(3,8)_ = 30.94, *P* < .0001) (Figure 3A, B, D, and E).

Levels of cleaved‐caspase 3 in the caudate and putamen were higher in the EP and EP‐sham‐DBS groups than in the control group, but were reduced in the EP‐DBS group in comparison with EP and EP‐sham‐DBS, suggesting that apoptosis was relieved by ANT‐DBS (caudate: control group 100.0 ± 16.0%, EP group 328.0 ± 72.2%, EP‐sham‐DBS group 373.1 ± 97.8%, EP‐DBS group 131.0 ± 43.8%, *F*_(3,8)_ = 13.39, *P* = .0017; putamen: control group 100.0 ± 13.2%, EP group 328.1 ± 76.9%, EP‐sham‐DBS group 344.3 ± 35.7%, EP‐DBS group 143.2 ± 45.2%, *F*_(3,8)_ = 20.04, *P* = .0004) (Figure 3A, C, D, and F).

### 3.5 Expression of GABA_A_‐R in GPi is increased by ANT‐DBS

**FIGURE 4:**
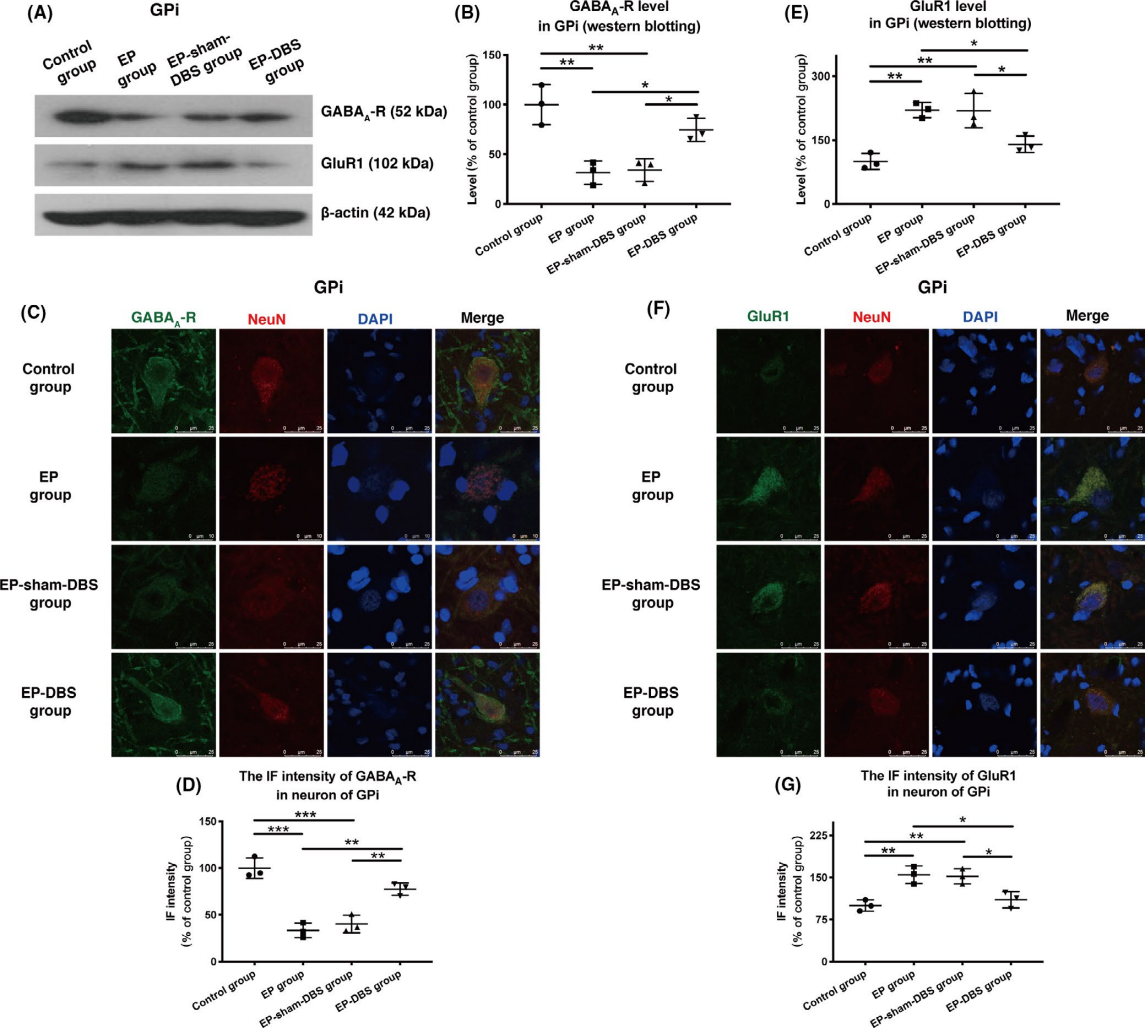
A, Analysis of GABA_A_‐R and GluR1 in the GPi via Western blotting. B, GABA_A_‐R was significantly reduced in the EP and EP‐sham‐DBS groups, but this was reversed by ANT‐DBS in the monkeys in the EP‐DBS group. C, Immunofluorescent staining of GABA_A_‐R and NeuN in the GPi. D, GABA_A_‐R was reduced in the TLE monkey model. Higher immunofluorescence intensity of GABA_A_‐R in GPi neurons was detected in the TLE monkey treated with ANT stimulation. E, Protein expression of GluR1 increased in the monkeys of the EP and EP‐sham‐DBS groups. Interestingly, the expression of GluR1 declined markedly in the GPi of the ANT‐DBS‐treated TLE monkeys. F, Immunofluorescent staining of GluR1 and NeuN in the GPi. G, More GluR1 was expressed in the GPi neurons of the EP and EP‐sham‐DBS groups. Expression of GluR1 decreased markedly in the GPi neurons of the EP‐DBS group. *P < .05; **P < .01; ***P < .001. DBS, deep brain stimulation; ANT, anterior nuclei of the thalamus; EP, epilepsy; GluR1, glutamate receptor 1 [Colour figure can be viewed at wileyonlinelibrary.com]

In normal monkeys, normal GABA_A_ receptor (GABA_A_‐R) levels were detected in the GPi. After kainic acid injection, significantly lower levels of GABA_A_‐R were observed in the EP and EP‐sham‐DBS groups. However, after ANT‐DBS, this reduction in GABA_A_‐R expression was reversed in monkeys of the EP‐DBS group relative to untreated monkeys with TLE (control group 100.0 ± 20.2%, EP group 31.5 ± 11.7%, EP‐sham‐DBS group 34.1 ± 11.4%, EP‐DBS group 74.5 ± 11.6%, F_(3,8)_ = 16.33, *P* = .0009) (Figure 4A,B).

GABA_A_‐R is expressed not only on neurons but also on glial cells and modulates glial cell functions.^18^ Therefore, GABA_A_‐R and NeuN were measured simultaneously in the GPi. The IF intensity for GABA_A_‐R in GPi neurons was lower for the TLE monkey model than for normal monkeys. However, relatively higher IF intensity for GABA_A_‐R was detected in GPi neurons of the TLE monkeys after ANT stimulation (control group 100.0 ± 11.0%, EP group 33.4 ± 7.8%, EP‐sham‐DBS group 40.2 ± 9.4%, EP‐DBS group 77.6 ± 60.6%, *F*_(3,8)_ = 38.04, *P* < .0001) (Figure 4C,D).

### 3.6 Expression of glutamate receptor in GPi is reduced by ANT‐DBS

GluR1 protein expression in GPi was elevated in monkeys of the EP and EP‐sham‐DBS groups, but ANT‐DBS treatment markedly reduced this expression (control group 100.0 ± 18.7%, EP group 220.4 ± 17.8%, EP‐sham‐DBS group 219.1 ± 40.3%, EP‐DBS group 140.3 ± 19.5%, *F*_(3,8)_ = 16.08, *P* = .0009) (Figure 4A,E).

Colocalization of NeuN and GluR1 was determined. Much more GluR1 (IF intensity) was expressed in single GPi neurons of TLE monkeys than in those of the control, but after ANT stimulation, its expression declined notably in the GPi neurons of the individuals in the EP‐DBS group. These results indicate that the glutamatergic projection to the GPi was downregulated by ANT‐DBS (control group 100.0 ± 10.0%, EP group 154.8 ± 15.7%, EP‐sham‐DBS group 152.0 ± 13.6%, EP‐DBS group 110.4 ± 14.6%, *F*_(3,8)_ = 12.79, *P* = .0020) (Figure 4F,G).

### 3.7 ANT‐DBS suppresses GAD‐67 expression in GPi

**FIGURE 5:**
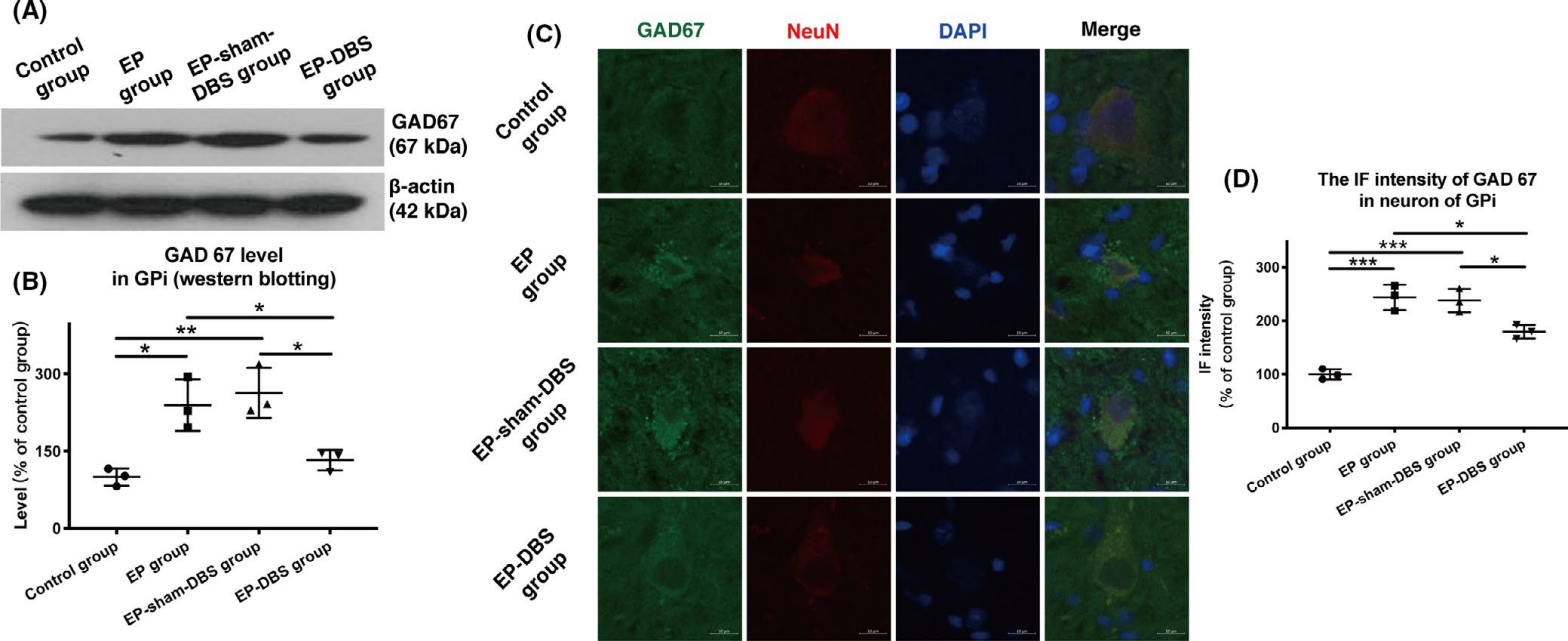
A, Analysis of GAD67 in the GPi via Western blotting. B, Expression of GAD67 was high in the GPi of the EP and EP‐sham‐DBS groups. A significant decline in GAD67 was observed in the TLE monkeys stimulated with ANT‐DBS in the EP‐DBS group. C, Immunofluorescent staining of GAD67 and NeuN in GPi. D, Immunofluorescent intensity of GAD67 increased in the TLE model, but was reversed by ANT‐DBS. *P < .05; **P < .01; ***P < .001. DBS, deep brain stimulation; ANT, anterior nuclei of the thalamus; EP, epilepsy; GAD67, glutamate decarboxylase 67 [Colour figure can be viewed at wileyonlinelibrary.com]

The Western blotting data for glutamate decarboxylase 67 (GAD67) showed normal levels in the GPi of the monkeys without TLE, but higher GAD67 expression in the GPi was detected in the EP and EP‐sham‐DBS groups. A reduction in GAD67 was noted in the TLE monkeys stimulated with ANT‐DBS in the EP‐DBS group (control group: 100.0 ± 16.8%; EP group: 239.6 ± 50.3%; EP‐sham‐DBS group: 263.3 ± 48.4%; EP‐DBS group: 132.7 ± 19.9%, *F*_(3,8)_ = 13.76, *P* = .0016) (Figure 5A,B). Consistent with the results described above, the colocalization of GAD67 and NeuN showed that the IF intensity of GAD67 increased in the TLE model, but was reversed by ANT‐DBS. These results indicate that the GPi projection was downregulated by ANT‐DBS (control group: 100.0 ± 9.7%; EP group: 243.8 ± 23.6%; EP‐sham‐DBS group: 237.9 ± 21.8%; EP‐DBS group: 179.6 ± 12.7%, *F*_(3,8)_ = 47.60, *P* < .0001) (Figure 5C,D).

## 4 DISCUSSION

The effects of ANT‐DBS on basal ganglia functions were investigated in an NHP‐TLE model. Our results indicate that ANT‐DBS downregulated overactivated GPi activity and exerted neuroprotective effects in the dorsal striatum, which may be associated with enhancement of the antiepileptic function of the basal ganglia that is dysfunctional in epilepsy (Table 1).

**TABLE 1:** Summary of changes in protein expression

	Control group	EP group	EP‐sham‐DBS group	EP‐DBS group
Caudate‐D1 receptor	→	↓	↓	↑
Caudate‐D2 receptor	→	↓	↓	↑
Putamen‐D1 receptor	→	↓	↓	↑
Putamen‐D2 receptor	→	↓	↓	↑
Caudate‐NeuN	→	↓	↓	↑
Caudate‐caspase‐3	→	↑	↑	↓
Putamen‐NeuN	→	↓	↓	↑
Putamen‐caspase‐3	→	↑	↑	↓
NAc‐GluR1	→	↑	↑	↓
GPi‐GABA_A_ receptor	→	↓	↓	↑
GPi‐GluR1	→	↑	↑	↓
GPi‐GAD67	→	↑	↑	↓

Abbreviations: GPi, globus pallidus internus; NAc, nucleus accumbens; GluR1, glutamate receptor 1; GAD67, glutamate decarboxylase 67.

### 4.1 Relationship between the limbic system and the basal ganglia

We used the NHP‐TLE model because anatomic differences between rodents and primates imply that functional differences exist that may influence the downstream pathways involved in seizure propagation.^19^, ^20^, ^21^ The hippocampus, amygdala, and ANT all belong to the limbic system and can be used as neuromodulatory targets.^3^, ^4^ Experimental and clinical data suggest that the NAc is involved in propagation of TLE and connects the limbic system with the basal ganglia circuits.^21^, ^22^ In detail, the ventral hippocampus sends glutamatergic projections to the NAc shell; it is noteworthy that this projection also extends to the NAc core in rodents, which does not occur in primates.^23^ The shell is the outer region of the NAc; it differs from the core and is considered to be part of the extended amygdala, an important part of the limbic system.^24^

In this study, increased glutamate receptor levels in the NAc shell were observed in the TLE model, but decreased after ANT‐DBS. GluR1 is closely associated with derangement of function in epileptogenesis.^25^, ^26^ The reduced excitatory toxicity directed against the NAc shell after DBS might be important for related structures in the basal ganglia, because NAc is a “gate.” Some may argue that increased glutamate receptor expression in the NAc during TLE is a secondary effect of hippocampal alteration. However, the hippocampal volume decreases when the excitatory input is increased, which makes it unlikely that it results from the increased afferent from the hippocampus.

### 4.2 Neural protective effect of ANT‐DBS on dorsal striatum (caudate and putamen)

The dorsal striatum has antiepileptogenic effects.^10^, ^27^ The seizure frequency in an amygdala‐kindled rodent model was reduced by injecting bicuculline (a GABA_A_‐R antagonist that enhances the dorsal striatum output) into the caudate‐putamen.^27^ Therefore, atrophy of the dorsal striatum might increase the frequency of epilepsy and its synchronization. In this study, apoptosis of basal ganglia neurons was increased in the NHP‐TLE model, which may explain the clinical neuroimaging finding that the volume of the dorsal striatum is reduced predominantly ipsilateral to the epileptic focus in TLE patients.^28^, ^29^ The present study also found that ANT‐DBS has a neuroprotective effect on the dorsal striatum, which may be the mechanism underlying its antiepileptic effect.

This hippocampal neuroprotective effect of ANT‐DBS has been demonstrated in many previous studies ^30^, ^31^; the underlying mechanisms proposed include inflammatory regulation and adenosine promotion.^16^, ^31^, ^32^ These results suggest that ANT‐DBS has an extensive effect by regulating these functions far from the stimulated area. Neuronal apoptosis in the dorsal striatum was increased in the TLE model, which might result from the excitatory toxicity of the limbic‐basal ganglia circuit. In this study, a neuroprotective effect was observed in the dorsal striatum after DBS, which confirms the more extensive neuroprotective effects of ANT‐DBS and might explain the alleviation of symptoms other than seizure by ANT‐DBS.^3^

### 4.3 Effect of ANT‐DBS on changes in dorsal striatum (caudate and putamen) D1 and D2 receptors in TLE

D1 and D2 receptors in the dorsal striatum are associated with epilepsy. A previous study also found that the dorsal striatum has antiepileptogenic effects.^10^, ^27^ D1 receptor levels are reduced in autosomal dominant nocturnal frontal‐lobe epilepsy.^33^ In TLE patients, D2 receptor levels are extensively reduced in striatal brain areas. One study demonstrated the protective effect of a D2 receptor agonist on pilocarpine‐induced epilepsy.^34^ Combined with the evidence reported above, this may indicate a correlation between the antiepileptic effect and changes in the basal ganglia. Some researchers also found that D2 receptor levels in the human dorsal striatum correlate negatively with the duration of epilepsy.^6^, ^35^

Several studies have shown that pretreatment with a D1 receptor agonist can induce convulsive activity after systemic administration. However, the extrastriatal dopamine system might also be involved in this process and therefore may have been a confounding factor.^36^ Another study showed that D2 agonists protect rodents against pilocarpine‐induced convulsions by stimulating D2 receptors in the striatum, but not those in the SNr.^34^ These findings indicate that the functions of the dopamine D1 and D2 receptors are highly location‐specific. Dopamine D1 and D2 receptors in the dorsal striatum might both be important in inhibiting seizures.

In this study, both D1 and D2 receptors were reduced in the dorsal striatum, which was the result of neuronal loss. ANT‐DBS can rescue neurons in the dorsal striatum and may exert antiepileptic effects in this way.

### 4.4 GPi activity changes in TLE and after ANT‐DBS

The SNr and GPi are both important output nuclei in the basal ganglia and send inhibitory projections.^37^ Because rodents do not have a GPi, previous studies have used the SNr instead and showed that inhibition of the SNr inhibits epileptogenesis.^8^, ^9^ Previous studies have also reported that high‐frequency DBS inhibits the STN or SNr and then activates the anticonvulsant zone of the dorsal midbrain.^38^, ^39^, ^40^ Therefore, in this study we focused on receptor changes in the GPi of a primate. We evaluated GluR1 and GABA_A_‐R in the GPi and found that GABA_A_‐R was reduced and GluR1 was increased in TLE, but these changes were partly reversed by ANT‐DBS.

The GPi mainly receives excitatory projections from the STN and inhibitory projections from the dorsal striatum.^37^ Therefore, loss of dorsal striatum neurons in the EP model could lead to overactivation of the GPi, whereas DBS would rebalance the excitatory and inhibitory inputs to the GPi by increasing the survival of dorsal striatal neurons. GAD67 catalyzes the decarboxylation of glutamate to GABA and therefore reflects the production of GABA. We also evaluated the inhibitory output of the GPi, which increased in the TLE model, as reflected in elevated GAD67. However, the inhibitory output of the GPi was reduced after DBS, which could improve the function of the anticonvulsant zone of the dorsal midbrain. Therefore, inhibition of the GPi might also be involved in the mechanism underlying the effect of ANT‐DBS, which suggests that stimulating the GPi may be a treatment option for epilepsy.

## 5 CONCLUSION

In conclusion, chronic ANT‐DBS exerts a neuroprotective effect on the dorsal striatum and reduces the loss of D1 and D2 receptors in the NHP‐TLE model. ANT‐DBS also downregulated the excessive activity of the GPi in the NHP‐TLE model, which may be associated with enhancement of the antiepileptic role of the basal ganglia. Our findings provide further evidence that normalization of basal ganglia function is involved in the molecular mechanism of ANT‐DBS therapy for TLE.

## CONFLICTS OF INTEREST

None.

## Supporting information

Figure S1

Table S1

## Data Availability

The data that support the findings of this study are available from the corresponding author upon reasonable request.
