# Supplementary material for: Deep brain stimulation of the anterior nuclei of the thalamus relieves basal ganglia dysfunction in monkeys with temporal lobe epilepsy
Source: CNS Neurosci Ther. 2020 Oct 21;27(3):341–51. doi: 10.1111/cns.13462 (PMC7871793; doi:10.1111/cns.13462)
Supplement: Supplementary file 1 — Figure S1 [file CNS-27-341-s001.pdf]

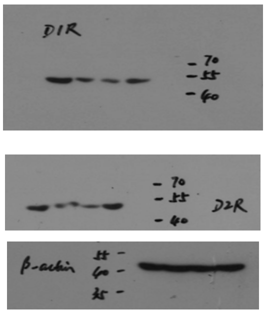

Full unedited gel/blot for Figure 1-Caudate

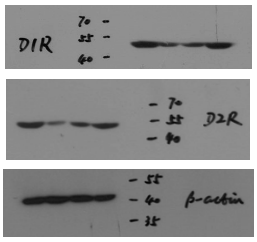

Full unedited gel/blot for Figure 1-Putamen

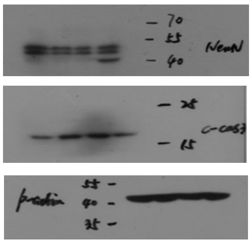

Full unedited gel/blot for Figure 3-Caudate

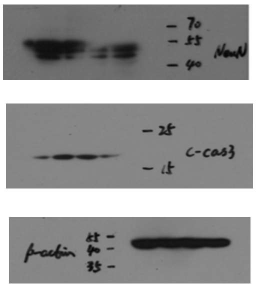

Full unedited gel/blot for Figure 3-Putamen

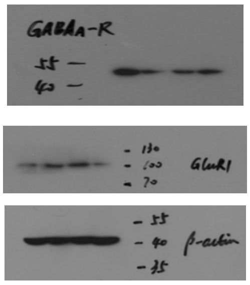

Full unedited gel/blot for Figure 4-GPi

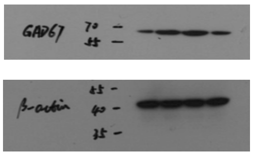

Full unedited gel/blot for Figure 5
