# Supplementary material for: Deep brain stimulation of the anterior nuclei of the thalamus relieves basal ganglia dysfunction in monkeys with temporal lobe epilepsy
Source: CNS Neurosci Ther. 2020 Oct 21;27(3):341–51. doi: 10.1111/cns.13462 (PMC7871793; doi:10.1111/cns.13462)
Supplement: Supplementary file 2 — Table S1 [file CNS-27-341-s002.docx]

**Supplementary Table 1.** *P* values for the different comparisons.

|  | Control vs EP group | Control vs EP-sham-DBS group | Control vs EP-DBS group | EP vs. EP-sham-DBS group | EP vs. EP-DBS group | EP-sham-DBS vs. EP-DBS group |
| --- | --- | --- | --- | --- | --- | --- |
| Number of D1 positive neurons in caudate | <0.0001 | <0.0001 | 0.0012 | >0.9999 | 0.0049 | 0.0190 |
| D1 receptor levels in caudate (western blotting) | 0.0064 | 0.0040 | >0.9999 | >0.9999 | 0.0151 | 0.0091 |
| Number of D2 positive neurons in caudate | 0.0002 | 0.0002 | 0.0696 | >0.9999 | 0.0053 | 0.0040 |
| D2 receptor levels in caudate (western blotting) | 0.0007 | 0.0011 | 0.1534 | >0.9999 | 0.0157 | 0.0338 |
| Number of D1 positive neurons in putamen | <0.0001 | <0.0001 | 0.0047 | >0.9999 | 0.0186 | 0.0163 |
| D1 receptor levels in putamen (western blotting) | 0.0039 | 0.0034 | 0.6137 | >0.9999 | 0.0451 | 0.0385 |
| Number of D2 positive neurons in putamen | 0.0004 | 0.0005 | 0.1001 | >0.9999 | 0.0106 | 0.0148 |
| D2 receptor levels in putamen (western blotting) | 0.0003 | 0.0004 | 0.042 | >0.9999 | 0.0169 | 0.0286 |
| IF intensity of GABA_A_-receptor in GPi neurons | <0.0001 | 0.0002 | 0.087 | >0.9999 | 0.0017 | 0.0051 |
| GABA_A_-receptor levels in GPi (western blotting) | 0.0022 | 0.0028 | 0.3562 | >0.9999 | 0.0357 | 0.0494 |
| IF intensity of GluR1 in GPi neurons | 0.0070 | 0.0096 | >0.9999 | >0.9999 | 0.0243 | 0.0344 |
| GluR1 levels in GPi (western blotting) | 0.0027 | 0.0029 | 0.5555 | >0.9999 | 0.0316 | 0.0344 |
| IF intensity of GAD67 in GPi neurons | <0.0001 | <0.0001 | 0.0037 | >0.9999 | 0.0141 | 0.0244 |
| GAD67 levels in GPi (western blotting) | 0.0106 | 0.0040 | >0.9999 | >0.9999 | 0.0473 | 0.0158 |
| IF intensity of GluR1 in NAc neurons | 0.0043 | 0.0041 | 0.7275 | >0.9999 | 0.0431 | 0.0404 |
| NeuN levels in caudate (western blotting) | 0.0012 | 0.0028 | 0.3777 | >0.9999 | 0.0158 | 0.0475 |
| Cleaved-caspase-3 levels in caudate (western blotting) | 0.0159 | 0.0053 | >0.9999 | >0.9999 | 0.0360 | 0.0112 |
| NeuN levels in putamen (western blotting) | 0.0001 | 0.0005 | 0.0426 | 0.9646 | 0.0049 | 0.0384 |
| Cleaved-caspase-3 levels in putamen (western blotting) | 0.0025 | 0.0016 | >0.9999 | >0.9999 | 0.0096 | 0.0057 |

GPi, globus pallidus internus; IF, immunofluorescence; NAc, nucleus accumbens; GluR1, glutamate receptor 1; GAD67, glutamate decarboxylase 67.
